# Confined and interface optical phonon emission in GaN/InGaN double barrier quantum well heterostructures

**DOI:** 10.1371/journal.pone.0214971

**Published:** 2019-04-18

**Authors:** Ahmed Mohamed, Kihoon Park, Can Bayram, Mitra Dutta, Michael Stroscio

**Affiliations:** 1 Department of Electrical and Computer Engineering, University of Illinois at Chicago, Chicago, Illinois, United States of America; 2 Department of Electrical and Computer Engineering, University of Illinois at Urbana-Champaign, Urbana, Illinois, United States of America; University of California Merced, UNITED STATES

## Abstract

In GaN-based high electron mobility transistors (HEMTs), the fast emission of longitudinal optical (LO) phonons can result in the formation of hot spots near the gate region where high electric fields produce hot electrons. In this work, we investigate the probability of phonon emission as a function of electron energy for confined and interface (IF) phonon modes for wurtzite GaN/InGaN/GaN heterostructures. Hot electrons radiate optical phonons which decay, anharmonically, into acoustic phonons that are essentially heat carriers. Herein, phonon engineering concepts are introduced which facilitate thermal management through the production of polar optical phonons. Some of the electrons near a semiconductor gate which manifests a strong electric field, are accelerated and the resulting hot electrons will produce confined and interface modes when the electrons are incident on a suitably-placed quantum well. This paper focuses on the production of confined and interface phonons. It is shown that interface modes may be preferentially produced which lead to elongated, lower-temperature hot spots.

## Introduction

III-nitride semiconductors are technologically important materials and as a result of their large bandgap energies, they are suitable for the optoelectronic intersubband devices ranging from the ultraviolet to the near infrared [1]. Nowadays, they are widely used, for instance in lighting applications, including GaN-based white light emitting diodes or in blue-ray players, which rely on GaN-based laser diodes. They can also endure high electric power densities and high breakdown voltages, which makes them materials of interest for high-power, high-frequency electronics applications [1,2].

GaN/InGaN multiple quantum wells (MQWs), with low In-content, offers tunable band gaps ranging from 2.9–3.4 eV for In content (0–0.4) [3]. They also have been well-regarded for their applications in quantum well infrared photodetectors (QWIPs), quantum cascade infrared photodetectors (QCIPs), and blue and white light emitting diodes. They are considered strong candidates for artificial lighting to replace the incumbent conventional fluorescent and incandescent lighting [2]. However, as nitride devices, and semiconductors in general, are being scaled down to operate in nanometer regions, reliability issues appear due to the heat generation and increase in junction temperature which contribute significantly to the degradation of device’s performance. In view of the advantages and applications of InGaN QWs, it is fitting to investigate the possibility of enhancing their reliability via optical phonon emission as phonon engineering emerges as an important tool in nanoscale regimes [4].

Studies have shown the importance of investigating the interaction between electrons and polar-optical-phonons, as polar optical phonon emission is known as the primary energy relaxation process of hot electrons in GaN [5,6]. The importance of carrier-phonon interactions and phonon-assisted processes is now well-known and is illustrated in a number of different nanostructures [7,8,9]. Polar optical electron-phonon scattering through the Frohlich interaction is the dominant scattering process in low-defect GaN/InGaN QWs over a wide range of temperatures; moreover, the optical phonon production dominates over the acoustic phonons production via the deformation potential [10].

In our analysis, we start by formulating the phonon total scattering rate for GaN/InGaN double heterostructure quantum well (QW). We consider an initial design of a 5 nm QW of a hexagonally ordered wurtzite In_0.15_Ga_0.85_N sandwiched between two other hexagon planes of GaN wurtzite. We examine the wave parameters, the dispersion relation and the group velocities of the available phonon modes and demonstrate that adjusting the thickness of the inserted InGaN layer can substantially impact the polar optical phonons emission rates.

### Optical phonon modes in GaN/ InGaN quantum wells

**Fig 1:**
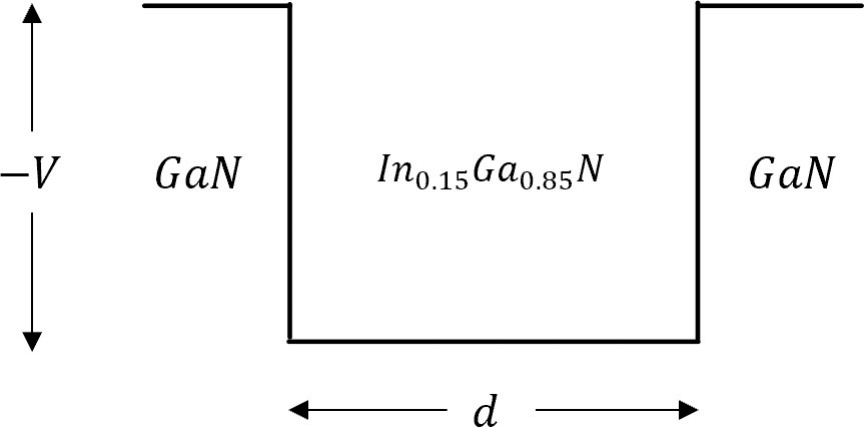
Schematic diagram of a typical GaN/InGaN/GaN double barrier quantum well structure. The width *d* of the InGaN layer is set initially to 5 nm for the scattering rates numerical calculations. The well depth *v* of 0.5 eV represents the discontinuity between the conduction bands of InGaN and GaN. The values reported for the conduction band energies (*E*_*C*_) are -2.8 eV and -2.3 eV for GaN and InGaN based on DFT calculations [12].

Fig 1 introduces a schematic for the case of a simplified layered-structure of GaN/In_0.15_Ga_0.85_N/GaN QW. The difference between the dielectric constants between GaN and InGaN leads to the production of two types of polar optical phonon modes: 1) interface phonon modes, which fall off evanescently from the heterointerface and, 2) confined phonon modes, which are confined in the heterostructure. In this paper, we calculated the scattering rates $\frac{1}{\tau }$ for these two phonon modes as functions of the electron energy E_k_ for GaN/In_0.15_Ga_0.85_N/GaN QWs, which are based on the analysis and the formalism of Ref. 11. It is shown that through phonon engineering, it is possible to preferentially channel the phonon emission into interface modes propagating with high optical phonon velocities which lead to elongated, lower-temperature hot spots in the region where the interface optical phonons decay into acoustic phonons.

**Fig 2:**
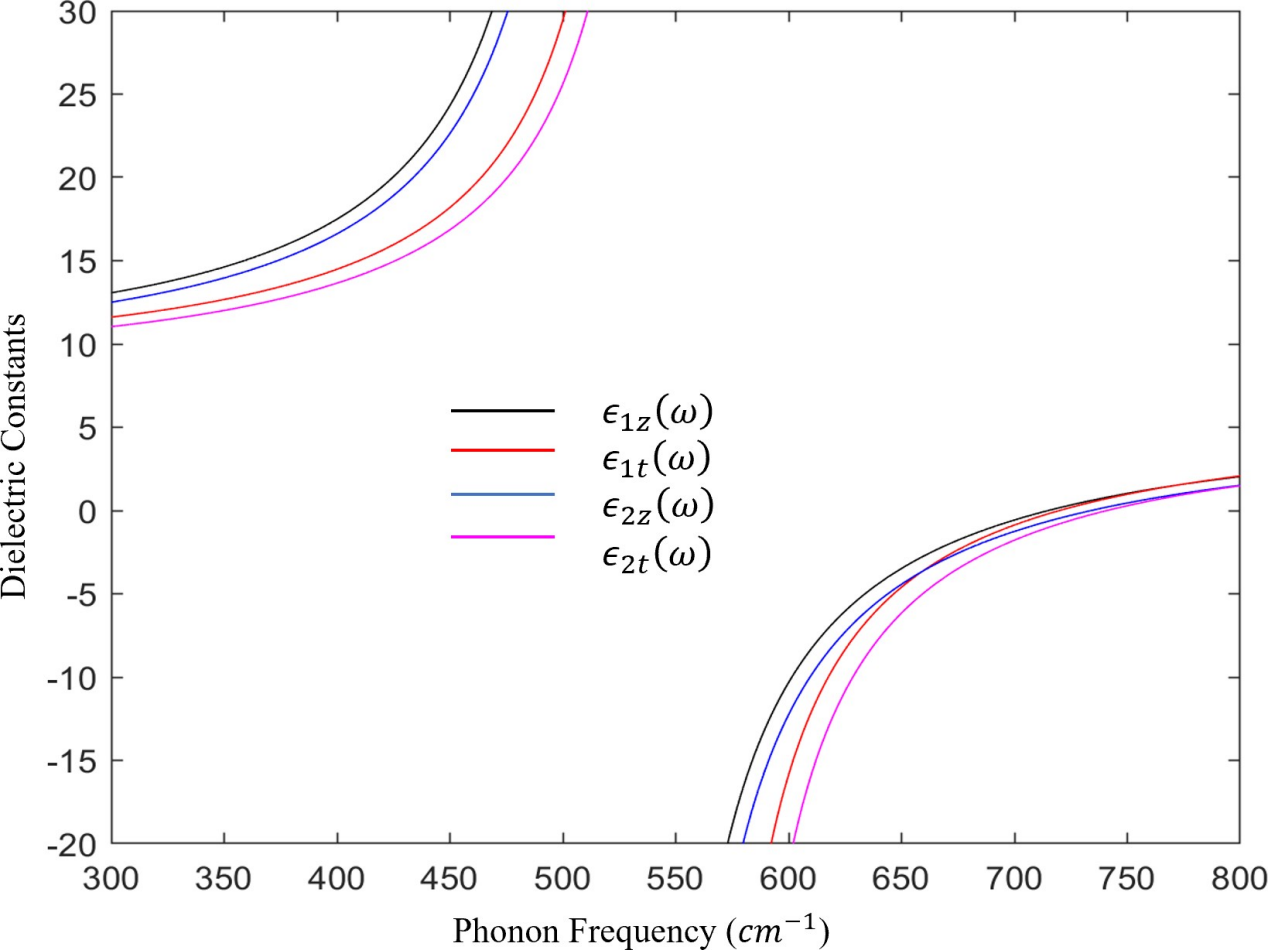
The frequency-dependent dielectric functions of GaN/InGaN/GaN QW. *ϵ*_1*z*_(*ω*) and *ϵ*_1*t*_(*ω*) for InGaN are designated in black and red and *ϵ*_2*z*_(*ω*) and *ϵ*_2*t*_(*ω*) for GaN are designated in blue and magenta.

Phonons of confined and interface modes coexist in certain regions in GaN/InGaN wurtzite heterostructures [13,14,15]. The range of the allowed frequencies is illustrated by the frequency-dependent dielectric constants [14,16,17]:

$$\epsilon_{1,2z}(\omega )=\epsilon_{z}^{\infty }\frac{\omega^{2}-\omega_{lz}^{2}}{\omega^{2}-\omega_{z}^{2}}$$ (1)

$$\epsilon_{1,2t}(\omega )=\epsilon_{t}^{\infty }\frac{\omega^{2}-\omega_{lt}^{2}}{\omega^{2}-\omega_{t}^{2}}$$ (2)

where *ω*_*lz*_, *ω*_*z*_, *ω*_*lt*_, and *ω*_*t*_ represent the characteristic frequencies of *A*_1_(*LO*), *A*_1_(*TO*), *E*_1_(*LO*), and *E*_1_(*TO*), respectively, and $\epsilon_{z}^{\infty }$ and $\epsilon_{t}^{\infty }$ are the optical dielectric constants for longitudinal and transverse modes, respectively. For GaN/InGaN with indium content of 15%, confined modes exist within two frequency intervals: *C*_*I*_ = [*ω*_1*z*_,*ω*_1*t*_] and *C*_*II*_ = [*ω*_1*lz*_,*ω*_1*lt*_], while interface modes exist between [*ω*_2*z*_,*ω*_1*t*_] and [*ω*_1*lt*_,*ω*_2*lz*_]. In this report, subscript 1 is assigned for InGaN and 2 is assigned for GaN. *ϵ*_1,2*z*_(*ω*) and *ϵ*_1,2*t*_(*ω*) are plotted as shown in Fig 2. The dielectric constants *ϵ*_1*z*_(*ω*) and *ϵ*_1*t*_(*ω*) in Fig 2 converge at large values near their TO frequencies of *ω*_*z*_ and *ω*_*t*_ for both materials. The dielectric constants switch to negative values outside the range of the allowed frequencies > 741 cm^-1^ and remain positive at values < 525 cm^-1^, which indicate that *ϵ*_1,2*z*_(*ω*)*ϵ*_1,2*t*_(*ω*)<0 always holds for GaN/InGaN structure. In that case, no overlap of the characteristic frequencies will occur, and the boundary conditions will satisfy neither the oscillating solution nor the decaying solution for the existence of IF and confined modes.

The phonon potentials inside and outside the QWs for confined modes are given by [10]

$$\Phi_{1}(z)=\Phi_{0}\cos\left(2\alpha q\frac{z}{d}\right)$$ (3)

$$\Phi_{2}(z)=A\;\cos\left(\alpha q\right)e^{\beta q(1-2\frac{z}{d})}$$ (4)

and for interface modes

$$\Phi_{1}(z)=A\;\cosh\left(2\alpha qz\right)$$ (5)

$$\Phi_{2}(z)=A\;\cosh\left(\alpha qd\right)e^{\beta qd(1-2\frac{z}{d})}$$ (6)

where $\alpha =\frac{1}{2}\sqrt{\left|\epsilon_{1t}/\epsilon_{1z}\right|},\;\beta =\frac{1}{2}\sqrt{\left|\epsilon_{2t}/\epsilon_{2z}\right|},$ *q* is the phonon wave vector, *z* is defined within −*d*/2≤*z*≤*d*/2, and Φ_0_ and *A* are the normalization constants determined from the continuity of the Frohlich potentials at the GaN/InGaN heterointerfaces.

Within the context of the dielectric continuum approximation and Loudon’s models for uniaxial crystals, the scattering rate by emission and absorption is calculated with Fermi’s golden rule based on perturbation theory. The equation is defined as

$$\frac{1}{\tau }=2e^{2}\int_{0}^{\pi }d\theta \times \int \left(N_{q}+0.5\mp 0.5)D(q,\omega )\delta (E_{k+q}-E_{k}\mp \hbar \omega \right)dq$$ (7)

where *E*_*k*+*q*_−*E*_*k*_∓ℏ*ω* represents energy conservation in the delta function. The upper sign is defined for absorption and the lower sign is for emission. The phonon potentials were used to obtain *D*(*q*,*ω*) which was derived for interface and confined modes using the form factor (f2F). *f* and *F* were defined for wurtzite materials as [11]

$$f=\frac{\Upsilon^{2}}{\sqrt{1+(\frac{\xi_{2}}{\xi_{1}})}}\left[\frac{\cos^{2}\left(k_{1}\frac{d}{2}\right)}{k_{2}+\beta q}+\frac{(2\cos^{2}\left(k_{1}\frac{d}{2}\right)\alpha^{2}q^{2}\mp k_{1}^{2})\mu \frac{\xi_{2}}{\xi_{1}}\mp \alpha qk_{1}\sin\left(k_{1}d\right)}{2\alpha q(\alpha^{2}q^{2}\mp k_{1}^{2})}\right]$$ (8)

and

$$F=\frac{1}{1+(\frac{\xi_{2}}{\xi_{1}})}\left[2\beta \frac{\partial \epsilon_{2z}}{\partial \omega }+\frac{1}{2\beta }\frac{\partial \epsilon_{2t}}{\partial \omega }\pm \mu \xi_{2}(\frac{1}{\left|\epsilon_{1t}\right|}\frac{\partial \epsilon_{1t}}{\partial \omega }\mp \frac{1}{\left|\epsilon_{1z}\right|}\frac{\partial \epsilon_{1z}}{\partial \omega })+\alpha qd(2\alpha \frac{\partial \epsilon_{1z}}{\partial \omega }\pm \frac{1}{2\alpha }\frac{\partial \epsilon_{1t}}{\partial \omega })\right]$$ (9)

therefore

$$D^{\left(\begin{array}{c} C\\ IF \end{array}\right)}(q,\omega )==\frac{{\left[\frac{\Upsilon^{2}}{\left(\alpha^{2}q^{2}\mp k_{1}^{2}\right)}[\cos^{2}\left(k_{1}\frac{d}{2}\right)(\frac{k_{1}^{2}\mp \alpha^{2}q^{2}}{k_{2}+\beta q}\xi_{1}-2\alpha q\mu \xi_{2})\pm \frac{k_{1}^{2}\mu \xi_{2}}{2\alpha q}+\frac{1}{2}k_{1}\xi_{1}\sin\left(k_{1}d\right)]\right]}^{2}}{{\xi_{1}}^{2}\xi^{+}(\beta )\pm \mu \xi_{1}\xi_{2}\xi^{\mp }(\alpha )+\alpha qd\xi^{\pm }(\alpha )(\xi_{1}^{2}\pm \xi_{2}^{2})}$$ (10)

Here, the upper sign is for confined phonons and the lower sign is for IF phonons. *k*_1_ and *k*_2_ are the parameters characterizing the electron wave vectors inside and outside the quantum well (in our case InGaN), respectively. They were obtained by solving Schrodinger’s wave equation for finite potential well for the ground state. The amplitude of the electron wave function is written as $\Upsilon ={\left[\frac{\cos\left(k_{1}d\right)\sin\left(k_{1}d\right)}{k_{1}}+\frac{d}{2}+\frac{\cos^{2}\left(k_{1}\frac{d}{2}\right)}{k_{2}}\right]}^{-1/2}$, with $\xi_{1}=\sqrt{\left|\epsilon_{1t}\epsilon_{1z}\right|},\;\xi_{2}=\sqrt{\left|\epsilon_{2t}\epsilon_{2z}\right|},\;\mu =\mathrm{sign}\left(\epsilon_{1t}\epsilon_{1z}\right),\;\xi^{+}(\beta )=2\beta \frac{\partial \epsilon_{2z}}{\partial \omega }+\frac{1}{2\beta }\frac{\partial \epsilon_{2t}}{\partial \omega }$ and $\xi^{\pm }(\alpha )=2\alpha \frac{\partial \epsilon_{1z}}{\partial \omega }\pm \frac{1}{2\alpha }\frac{\partial \epsilon_{1t}}{\partial \omega }$

To further understand the electron-phonon scattering process, let us look at the matrix element for the excitation of electron from a state *E*_*k*_ to *E*_*k*+*q*_. To describe the probability of a transition of an electron with wavevector *k* in the subband by a phonon of wavevector *q*, the matrix element can be defined as

$${\left|M^{\left(\begin{array}{c} C\\ IF \end{array}\right)}(q,\omega )\right|}^{2}=\frac{C(N(\omega )+\frac{1}{2}\mp \frac{1}{2})D(q,\omega )[(\frac{\omega }{q^{2}}\pm \frac{\hbar }{2m})\frac{\partial q}{\partial \omega }-\frac{1}{q}]}{q(\frac{q}{2}\pm \frac{m}{\hbar }[\frac{\omega }{q}-\frac{\partial \omega }{\partial q}])\sqrt{\frac{1}{m}(2E_{k}-\frac{1}{2}E_{q}\pm \hbar \omega )-\frac{\omega^{2}}{q^{2}}}}$$ (11)

where *C* is a constant expressed in Kilogram.Ampere (Kg.A). The largest scattering probability for small *q* can be justified by the 1/*q* dependence in Eq (11). We are interested in small *q* values as they contribute significantly to the Frohlich interaction above the phonon emission threshold when phonons strongly interact with electrons in the GaN/InGaN heterostructure, thus creating phonons along the heterointerface which combine mixing of LO and TO modes. Eq (11) conserves both energy and momentum whereas the angle dependence was removed by expressing *dθ* as $\frac{d\theta }{d\omega }d\omega$ when employing

$$\cos\;\theta =\pm \frac{m^{*}m_{0}\omega }{kq\hbar }-\frac{q}{2k}$$ (12)

The formalism of obtaining the electron-phonon scattering rates is based on integrating over the energy conversion delta function. For the case of emission, the scattering rate equation is finally expressed as

$$\frac{1}{\tau^{\left(\begin{array}{c} a\\ e \end{array}\right)}}=\pm \frac{2m^{*}}{a_{B}}\sum_{n}\int_{\omega 1}^{\omega 2}\frac{\left(N(\omega )+\frac{1}{2}\mp \frac{1}{2})D(q,\omega )[(\frac{\omega }{q^{2}}\pm \frac{\hbar }{2m})\frac{\partial q}{\partial \omega }-\frac{1}{q}\right]}{(\frac{q}{2}\pm \frac{m}{\hbar }[\frac{\omega }{q}-\frac{\partial \omega }{\partial q}])\sqrt{\frac{1}{m}(2E_{k}-\frac{1}{2}E_{q}\pm \hbar \omega )-\frac{\omega^{2}}{q^{2}}}}d\omega$$ (13)

where $a_{B}=\frac{\hbar^{2}}{m_{0}e^{2}}$ is defined as the Bohr radius in free space, *n* is an integer which represents the quantum number of phonons in the field and $N(\omega )=\frac{1}{exp(\frac{\hbar \omega }{kT})-1}$ is the phonon occupation number with the energy of a single phonon being ℏ*ω*. The integration variable is the electron energy of the lowest subband given by $E_{k}=\frac{\hbar^{2}k^{2}}{2m}$. The phonon’s phase velocity (*v*_*ph*_) and group velocity (*v*_*gr*_) are expressed as $\frac{\omega }{q}$ and $\frac{\partial \omega }{\partial q}$, respectively. The group velocity is found numerically and discussed for IF and confined cases later on this paper. Here, $E_{q}=\frac{\hbar^{2}q^{2}}{2m}$ is a collection of the phonon’s wave number, Planck’s constant and the electron’s effective mass, *m* = m^*^m_0_.

Eq (13) defines the case for both confined and interface phonon scattering rates, with the summation over *n* in the IF case is omitted [11]. The integral in Eq (13) is an integration over the range of allowed phonon frequencies [*ω*_1*z*_,*ω*_2*lz*_], indicated by *ω*_1_ to *ω*_2_ in the integral, as an independent variable. The integration limits are determined from the relation in Eq (12), however, the practical limits of the numerical integration are the allowed range of frequency for the phonon mode in question. The phonon dispersion relation, nevertheless, must be considered in the integral in Eq (13).

It is observed that the LO and TO phonon frequencies in GaN/InGaN QW vary in a single path from GaN to InGaN (defined as the one-mode behavior) [18]. For the numerical calculations, we assume that $\epsilon_{z}^{\infty }=\epsilon_{t}^{\infty }$ [10]. We also define In_0.15_Ga_0.85_N well of depth 0.5 eV (since Δ*E*_*GaN*−*InGaN*_ = 0.5 *eV*), and the electron effective mass which is obtained through direct linear interpolation using Vegard’s law [19]. Also, due to the limited In-content, we found that using the effective mass parameter of GaN exhibits similar scattering rates performance. The c-axis of our heterostructure is aligned with the z-axis orientation and perpendicular to the interface. Table 1 summarizes the values used for the material constants [10,11,18].

**Table 1:** Material constants used in the present calculations for wurtzite GaN/InGaN/GaN QW.

Material	*ω*_*lz*_(*cm*^−1^)	*ω*_*z*_(*cm*^−1^)	*ω*_*lt*_(*cm*^−1^)	*ω*_*t*_(*cm*^−1^)	*ϵ*^∞^
In_0.15_Ga_0.85_N	715	525	720	550	5.76
GaN	734	531	741	559	5.35

### Dispersion relations

The dispersion relations for the IF and confined modes are given by

$$q=\frac{1}{2}\ln\left[\frac{\xi_{1}+\xi_{2}}{\pm (\xi_{1}-\xi_{2})}\right]/\left(\alpha d\right)$$ (14)

and

$$q_{n}=\left[n\pi +\mu \arctan\left(\xi_{2}/\xi_{1}\right)\right]/\left(\alpha d\right)$$ (15)

and

$$n=1,2,3,\ldots \;\mathrm{and}\;0\;\mathrm{if}\;\mu =1q_{n}=[n\pi -\mu \arctan\left(\xi_{1}/\xi_{2}\right)]/(\alpha d)$$ (16)

n=1,2,3,…and0ifμ=−1

**Fig 3:**
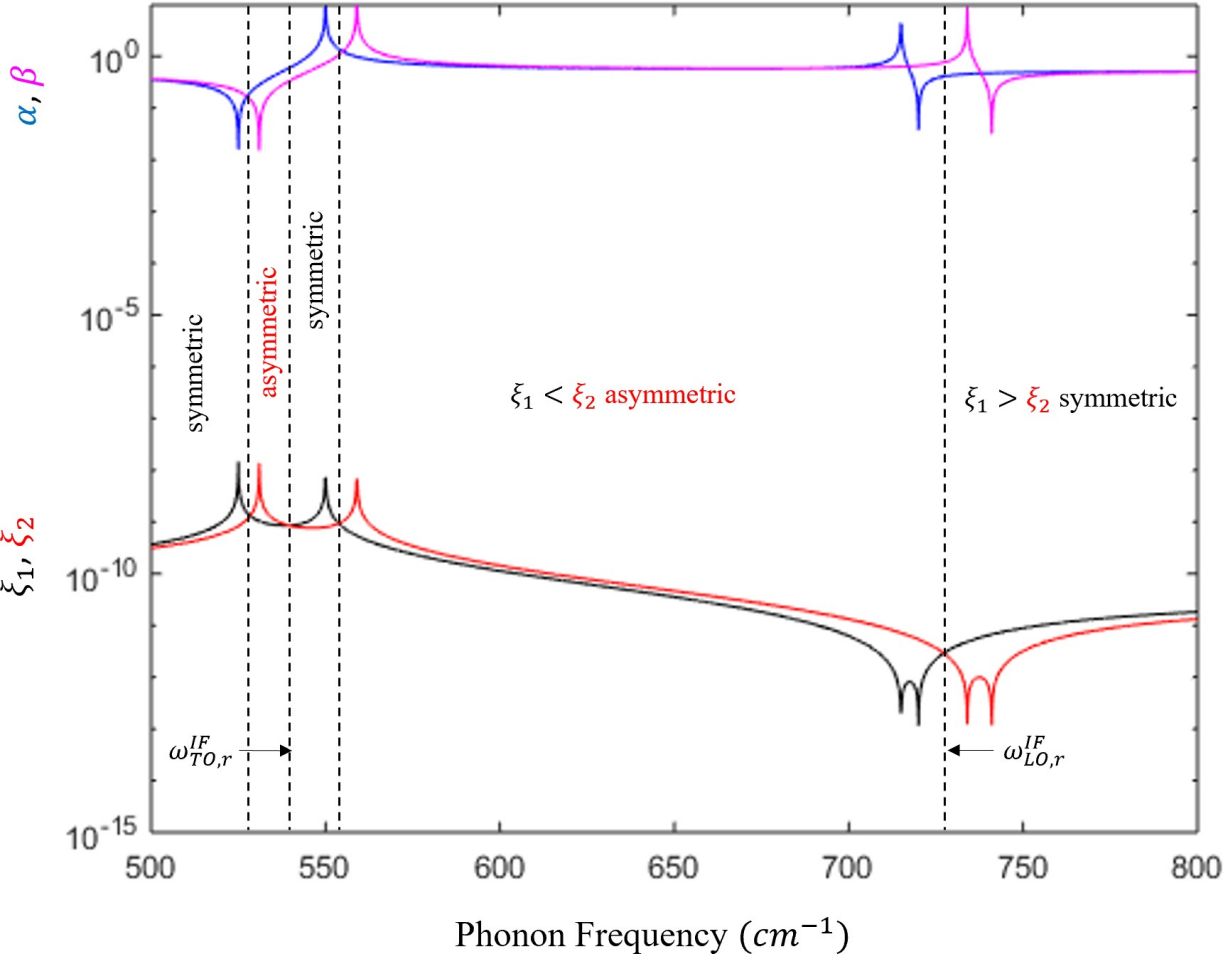
Parameters of the phonon wave function. *ξ*_1_ and *ξ*_2_ are indicated by black and red and *α* and *β* are indicated by blue and magenta. The vertical black dotted lines separate each symmetric and asymmetric region.

**Fig 4:**
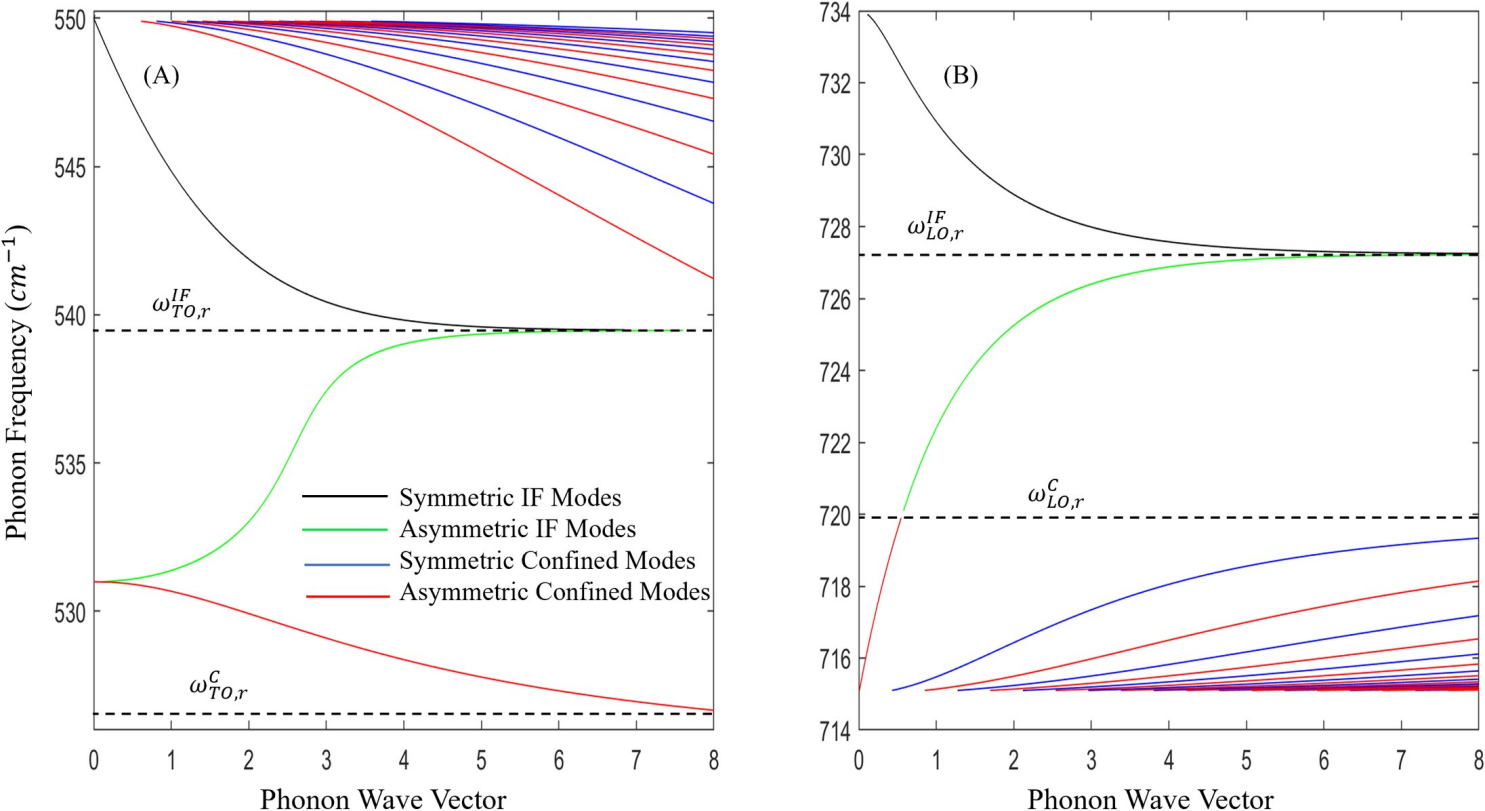
Dispersion relation of frequencies ℏ*ω* in a wurtzite GaN/In_0.15_Ga_0.85_N/GaN quantum well as a function of free phonon wave number when *n* = 0. The resonant frequency for IF and confined are indicated by the black dotted lines. The modes depicted are symmetric IF in black, asymmetric IF in green, even confined in blue and odd confined in red. The low-order energy intervals ℏ*ω*_*TO*_ are shown in Fig 4A and the high-order energy intervals ℏ*ω*_*LO*_ are shown in Fig 4B.

Eq (14) represents the case for IF modes. The upper sign in the denominator is for symmetric modes and the lower sign is for asymmetric modes. Eq (15) is defined for even confined modes and Eq (16) is defined for odd confined modes. The symmetric and asymmetric phonons satisfy the relations *ξ*_1_>*ξ*_2_ and *ξ*_1_<*ξ*_2_, respectively. Indeed, these relations were illustrated in Fig 3. The characteristic frequencies can be obtained from the peaks of *ξ*_1_ and *ξ*_2_ while their intersections denote the resonant frequencies. Fig 4A and Fig 4B depict the dispersion of polar optical phonons for interface and confined modes for low and high energy regions. The symmetric IF modes are shown in black and the asymmetric modes are shown in green. Also, the even confined modes are plotted in blue and the odd confined modes are plotted in red. The dispersive behavior of Eqs (15) and (16) shows that the confined modes usually have infinite solutions for given *n* and *q* in the intervals [*ω*_1*z*_,*ω*_1*t*_] and [*ω*_1*lz*_,*ω*_1*lt*_], however, only a certain number of confined modes are considered at given *n* and *q*. It is evident from the dispersion curves that the scattering rates increase due the strong presence of the confined phonons. From Fig 4, one can see that only the first few confined modes for any odd and even modes are considered. The higher order modes are normally ignored because 〈*f*|*v*|*i*〉≈0, when the potential *v* has many oscillations. The IF modes of lower frequencies associated with TO modes propagate around a resonant frequency of 539.5 cm^-1^, defined as $\omega_{TO,r}^{IF}$ in Fig 4, whereas the higher frequencies (LO modes) have a resonant frequency of 727.2 cm^-1^, defined as $\omega_{LO,r}^{IF}$ in Fig 4. As *q* approaches ∞, the confined modes saturate at 525.1 cm^-1^ (~*ω*_1*z*_) and 719.9 cm^-1^ (~*ω*_1*lt*_), which are labeled as $\omega_{TO,r}^{C}$ and $\omega_{LO,r}^{C}$ to show that they denote the emission thresholds for confined modes at the low and high frequency regions, respectively. The confined modes asymptotically reduce to the characteristic frequencies *ω*_1*t*_ for TO modes and *ω*_1*lz*_ for LO modes. The trend verifies that the confined modes exist between [*ω*_1*t*_,*ω*_1*z*_] and [*ω*_1*lt*_,*ω*_1*lz*_]. The strong dependence of the scattering rates on the dispersion relation and the values of the above resonance frequencies help us predict the energy required to emit TO-like and LO-like phonons. In Fig 4, the slopes of the optical branches indicate which modes have higher group velocity. Nonetheless, the slopes of these optical phonons (including IF) are still slower than the acoustic phonons which open the door for our optical phonon engineering methodology, that there exist the possibility of carefully treating these branches to take advantage of their dominance over the acoustic modes.

## Discussion

**Fig 5:**
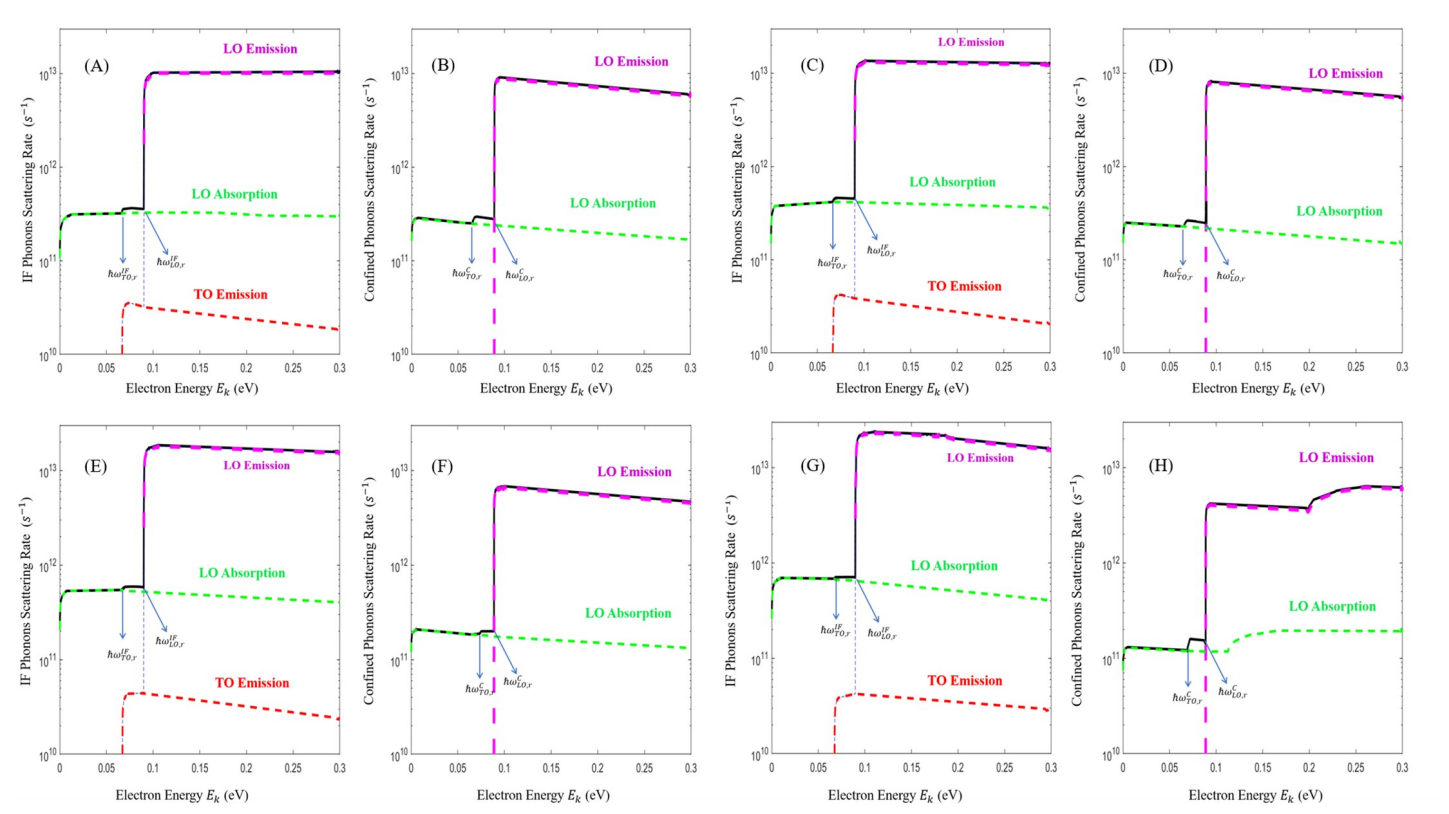
Total emission electron scattering rate as a function of the electron energy in the three-layered heterostructure of wurtzite GaN/InGaN/GaN quantum wells. The case *d = 5* nm is shown for interface in Fig 5A and confined in Fig 5B. The thickness is set to 4nm in Fig 5C and Fig 5D, 3 nm in Fig 5E and Fig 5F and 2 nm in Fig 5G and Fig 5H. Only the phonon-assisted emission for IF (Fig 5A, 5C, 5E and 5G) and confined modes (Fig 5B, 5D, 5F and 5H) is considered. The black solid line corresponds to the total emission rate which is the sum of the IF LO emission, LO absorption and the TO emission rates. For both symmetric and asymmetric emission, the magenta line corresponds to LO emission rates, the green line corresponds to LO absorption rates, the red line corresponds to TO emission rates. The TO emission threshold energies are indicated by blue arrows and are labeled in the graphs as $\hbar \omega_{TO,r}^{C}$ for confined and $\hbar \omega_{TO,r}^{IF}$ for IF while the LO emission threshold energies are labeled as $\hbar \omega_{LO,r}^{C}$ for confined and $\hbar \omega_{LO,r}^{IF}$ for IF.

Numerous studies have discussed the growth and the detection of lattice-matched GaN/InGaN QWs with well thicknesses as low as 1nm [20,21]. In this paper, it is shown that inserting a GaN/In_0.15_Ga_0.85_N/GaN quantum well in the hot spot region of a GaN HEMT, results in the emission of confined and interface phonons instead of bulk phonons as a result of emission from hot electrons, moreover, the phonons can be engineered to enhance the production of fast-moving interface phonons. Indeed, the IF and confined modes emission rates of GaN/In_0.15_Ga_0.85_N/GaN heterostructure with In_0.15_Ga_0.85_N thickness of *d* = 5, 4, 3, and 2 nm are numerically calculated and plotted in Fig 5. The thicknesses included in this paper allow us to grow GaN/InGaN QWs without misfit dislocations since they are below the critical thickness values [22]. In Fig 5, the left curves denote the IF scattering rates and the right curves denote the confined scattering rates. The total emission rate is presented in black which encompasses the LO emission in magenta, the LO absorption in green and the TO emission in red whereas the TO absorption is disregarded since it has a value well below 10^10^ s^-1^. These rates include both symmetric and asymmetric phonon process. The emission of TO-like and LO-like modes causes the first and the second step-like features in the black curves respectively. In the *d* = 5 nm case, hot electrons produce less confined phonons in GaN/InGaN QWs compared to GaN/AlN [17], this is referred to the weak confinement of phonons due to the lower potential barrier of the former QW.

In the hexagonal structure of GaN/InGaN, the medium anisotropy causes the splitting of LO and TO modes. Compared to the emission of LO phonons which has the highest scattering rates in the order of 10^13^ s^-1^, the emission of the TO phonons is much lower and in the order of 10^10^ to 10^11^ s^-1^ due to their weak coupling to electrons, thus the contribution from the TO modes is normally neglected. As can be seen from the graphs, the emission of LO (TO) phonons starts when the energy of the electron reaches a value right before ℏ*ω*_*LO*_ (ℏ*ω*_*TO*_). From the analysis of the IF (confined) resonance frequencies, we should predict LO phonons to be emitted at 90.16 meV (89.25 meV) and TO phonons to be emitted at 66.89 meV (65.1 meV). The values of *E*_*k*_ required to emit LO and TO phonons are illustrated on Fig 5 by $\hbar \omega_{LO,r}^{IF}=0.092\;eV,\;\hbar \omega_{TO,r}^{IF}=0.066\;eV$ for IF modes and $\hbar \omega_{LO,r}^{C}=0.089\;eV,\;\hbar \omega_{TO,r}^{C}=0.065\;eV$ for confined modes. We find that the values portrayed in Fig 5 are in close proximity to the resonant values.

When the InGaN thickness is set to 5 nm, IF (confined) emission rate reaches 1.13×10^13^ *s*^−1^ (9.75×10^12^ *s*^−1^) which means that approximately 53% of the hot electron energy is emitted as IF phonons. A modest increase (decrease) occurs with a QW thickness of 4nm to a maximum value of 1.45×10^13^ *s*^−1^ (8.345×10^12^ *s*^−1^), i.e., an increase in the probability of emitting IF phonons relative to confined phonons to about 63%. Following the analysis as mentioned, we find emission rates for IF (confined) approaching values of 1.89×10^13^ *s*^−1^ (6.83×10^12^ *s*^−1^) and 2.4×10^13^ *s*^−1^ (4.35×10^12^ *s*^−1^) for 3 and 2 nm thicknesses, respectively. From the trends of $\frac{1}{\tau }$ in Fig 5, the highest emission rates for confined phonons are located around their resonant frequencies but are becoming more difficult to produce in smaller QW thicknesses; hence one expects that these rates should be positioned at the onset of LO emission around *E*_*k*_ = 89.25 meV. Similarly, IF phonons are generated in greater numbers within the available frequency ranges. Therefore, presumably, the IF phonons that help dissipate heat more efficiently have energies above the emission threshold at *E*_*k*_ = 90.16 meV.

**Fig 6:**
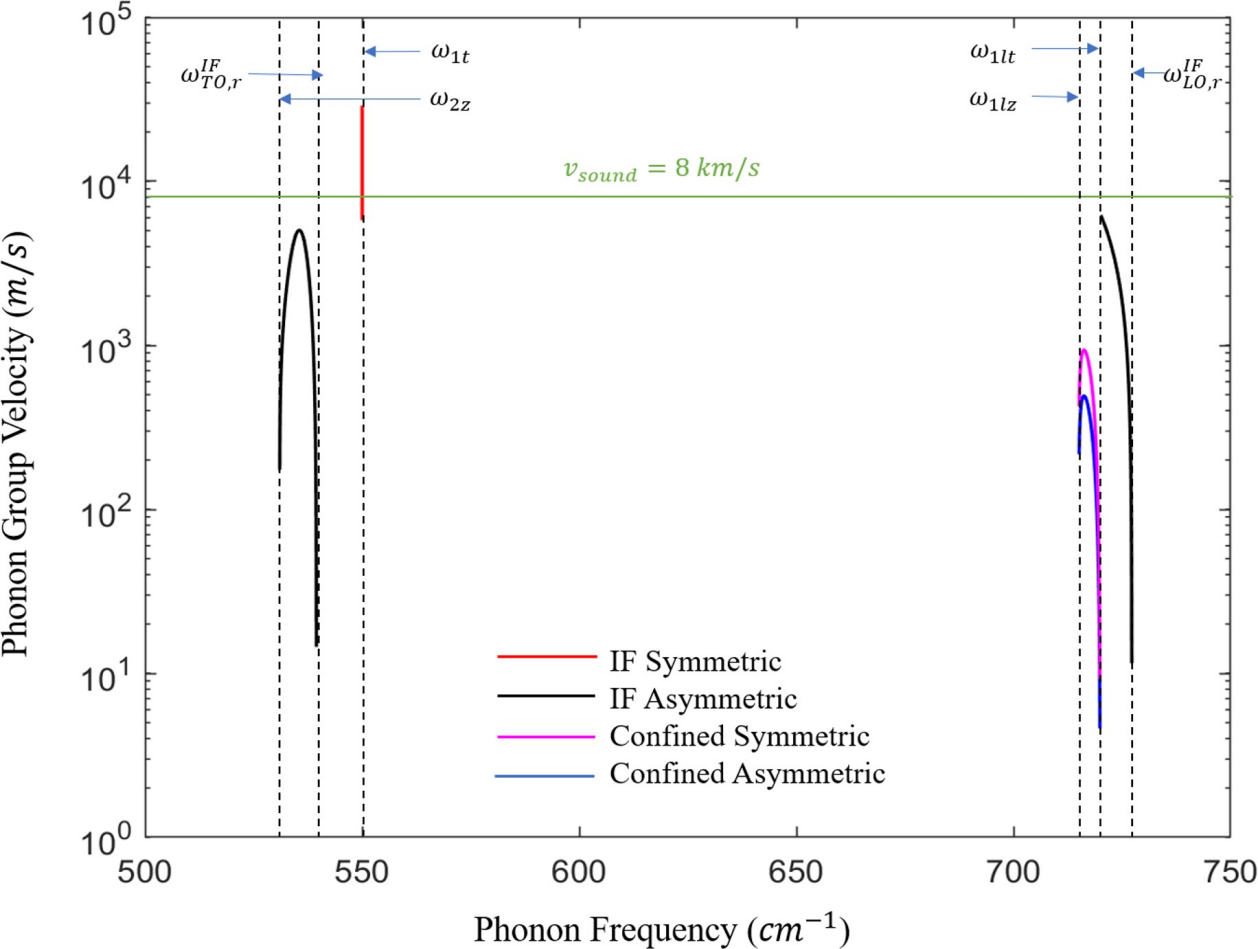
The group velocities of the IF and confined phonons against the phonon frequencies for the case of InGaN well of *d =* 5 nm. Only the modes in the intervals [*ω*_2*z*_,*ω*_1*t*_] and $\left[\omega_{1lz},\omega_{LO,r}^{IF}\right]$ which show significant group velocities are plotted. The symmetric IF (confined) phonons are plotted in red (magenta) and the asymmetric IF (confined) phonons are plotted in black (blue). For both modes, the group velocity is referenced to the acoustic sound velocity highlighted in green. Only the first confined mode is considered in the LO region for each symmetric and asymmetric mode. The resonance frequencies along with the system’s characteristic frequencies are shown by the horizontal blue arrows.

Normally, optical phonons are not considered as heat carriers as they have smaller group velocities compared to the acoustic phonons. As can be found in Fig 6, the group velocities of the optical modes in the interval [*ω*_2*z*_,*ω*_1*t*_] with *d =* 5 nm are noticeable and surprisingly the group velocity of the low-order optical branches approaches that of the acoustic branches (*v*_*sound*_ = 8 *km*/*s*). We note that even the highest frequency optical phonons are significantly dispersive. The group velocity *v*_*gr*_ of the highest confined mode is 0.936 km/s as it is clear from Fig 6. This mode oscillates at 716.5 cm^-1^ and reduce to a minimum value near its resonance frequency of 719.9 cm^-1^. On the other hand, IF asymmetric-type phonons (denoted by black lines) propagate at $\left[\omega_{2z},\omega_{TO,r}^{IF}\right]$ and $\left[\omega_{1lt},\omega_{LO,r}^{IF}\right]$. A sharp increase occurs in their group velocities where they move with speeds near the sound velocity and reduce to minimum values at the resonance frequencies. The highest IF symmetric-type phonon (denoted by red line) approaches a velocity of 29 km/s at 550 cm^-1^. Going back to Fig 5A, the added layer of InGaN produces IF phonons with rates that are ~ 2.5 times larger than the confined phonons rates. We observe at smaller widths that the emission of IF modes dominates over confined. Of course, there is a tradeoff between increasing $\frac{1}{\tau }$ with decreasing *d* as *v*_*gr*_ drops accordingly. Moving from *d* = 5 *nm* to *d* = 2 *nm*, the probability of emitting IF phonons increases by ~31% to almost 84%, while *v*_*gr*_ drops by a factor of ~ 2.5 for each mode plotted in Fig 6. Engineering IF with high group velocity becomes most useful when we have comparable IF and confined emission rates such as the case in AlN/GaN/AlN QW. Compared to the increasing IF emission rates in GaN/InGaN/GaN, the average probability of IF emission in AlN/GaN/AlN with GaN thickness of 5, 4 and 3 nm was found to be between 50–55%. Therefore, we must prioritize the consideration of engineering *v*_*gr*_ to become as high as possible in order for IF optical phonons to reduce the junction temperature, consequently they can propagate at high relative speeds away from the hot spot before they decay in picoseconds into heat-carrying phonons.

Thus far, we have considered the energy loss rates by phonon emission. Optical phonon confinement has an effect on the phonon linewidth, the frequency shift observed through Raman scattering and the phonon lifetimes, but the confinement-related change in lifetime is generally no more than about a factor of 2 [23]. Evidently, the IF phonon lifetime is important to determine their contribution to heat generation in the semiconductor channel. For GaN, it was reported that A_1_(LO) mode decays into a lower energy optical mode and an acoustic mode via the Ridley channel after some time 0.1 ps. Since the E_1_(LO) mode is polarized along the quantum well interfaces–as opposed to normal to the quantum well interfaces as for the A_1_(LO) mode–the production of E_1_(LO) modes dominates for the case where the propagating component of the electron wavefunction is parallel to the heterointerfaces as modeled in this calculation; it is known that the E_1_(LO) has a lifetime of 3.34 ps [24]. From the calculations obtained for the group velocities in Fig 6, we observe that different modes contribute to the size of the hot spot. For the E_1_(LO) mode, the asymmetric mode, labeled in black color in Fig 6, propagates with group velocity *v*_*gr*_ = 5×10^3^ *m*/*s*, this results in a spreading of the hot spot to approximately 17 nm. Similarly, the asymmetric mode in the high energy interval with *v*_*gr*_ = 6.2×10^3^ *m*/*s* extends the hot spot by about 21 nm. The highest velocity symmetric mode labeled in red in Fig 6 extends the hot spot by the largest amount of ~ 97 nm. These distances are very large compared to the hot spot sizes of a few nm observed in bulk GaN HEMTs [25].

## Conclusion

The frequency-dependent electron energy relaxation rates are calculated for GaN/InGaN/GaN quantum wells for different well widths. We have calculated the total scattering rate which includes both emission of IF and confined polar optical modes. It is shown that by quantum engineering of the inserted GaN/InGaN/GaN QW that the dominant phonon emission channel is that of interface phonon emission. Based on the large group velocity of the IF modes, as can be seen by comparing the IF phonon emission rates with confined phonon emission rates, a substantial fraction of the hot electron energy radiated as phonons goes into the IF phonon channel. Moreover, the IF propagate at high velocities compared to the bulk phonon velocities of the HEMT without the inserted QW. Accordingly, the anharmonically emitted heat-carrying acoustic phonons are generated in reduced-temperature, elongated hot spots. In summary, quantum engineering of IF phonon modes offers a means of thermal management of the hot spot temperature.
