# Correction: Confined and interface optical phonon emission in GaN/InGaN double barrier quantum well heterostructures

**DOI:** 10.1371/journal.pone.0216630

**Published:** 2019-05-02

**Authors:** The PLOS ONE Staff

In the Dispersion relations section, there is an error in Eq (16). The publisher apologizes for this error. Please view the original Eqs (14) and (15), and the corrected Eq (16) here:

$$q=\frac{1}{2}\ln\left[\frac{\xi_{1}+\xi_{2}}{\pm (\xi_{1}-\xi_{2})}\right]/\left(\alpha d\right)$$ (14)

and

$$q_{n}=\left[n\pi +\mu \;\arctan\left(\xi_{2}/\xi_{1}\right)\right]/\left(\alpha d\right)$$ (15)

*n* = 1,2,3,… and 0 if *μ* = 1

$$q_{n}=\left[n\pi -\mu \;\arctan\left(\xi_{1}/\xi_{2}\right)\right]/\left(\alpha d\right)$$ (16)

*n* = 1,2,3,… and 0 if *μ* = −1
